# Factors influencing consumer dietary health preventative behaviours

**DOI:** 10.1186/1471-2458-6-222

**Published:** 2006-09-01

**Authors:** Dan A Petrovici, Christopher Ritson

**Affiliations:** 1 Kent Business School, University of Kent, Parkwood Road, Canterbury, Kent, CT2 7PE, UK; 2 School of Agriculture, Food and Rural Development, Agriculture Building, 323 AB, University of Newcastle, Newcastle upon Tyne, NE1 7RU, UK

## Abstract

**Background:**

The deterioration of the health status of the Romanian population during the economic transition from a centrally planned to a free market economy has been linked to lifestyles factors (e.g. diet) regarded as a main determinants of the disparity in life expectancy between Eastern and Western Europe. Reforms in the health care system in this transition economy aim to focus on preventive action. The purpose of this study was to identify the factors that impact on the individual decision to engage in Dietary Health Preventive Behaviour (DHPB) and investigate their influence in the context of an adapted health cognition model.

**Methods:**

A population-based study recruited 485 adult respondents using random route sampling and face-to-face administered questionnaires.

**Results and discussion:**

Respondents' health motivation, beliefs that diet can prevent disease, knowledge about nutrition, level of education attainment and age have a positive influence on DHPB. Perceived barriers to healthy eating have a negative impact on alcohol moderation. The information acquisition behaviour (frequency of reading food labels) is negatively predicted by age and positively predicted by health motivation, education, self-reported knowledge about nutrition and household financial status. A significant segment of respondents believe they are not susceptible to the elicited diseases. Health promotion strategies should aim to change the judgments of health risk.

**Conclusion:**

The adaptation of the Health Belief Model and the Theory of Health Preventive Behaviour represents a valid framework of predicting DHPB. The negative sign of perceived threat of disease on DHPB may suggest that, under an income constraint, consumers tend to trade off long-term health benefits for short-term benefits. This cautions against the use of negative messages in public health campaigns. Raising the awareness of diet-disease relationships, knowledge about nutrition (particularly sources and risks associated with dietary fat and cholesterol) may induce people to adopt preventive dietary habits.

## Background

Cardiovascular diseases (CVD) represent the main cause of mortality in Romania, followed by cancer [1]. Unlike Western Europe, the mortality from CVD has increased steadily since the 1960s [2]. The mortality rates from cancer have almost doubled in Romania over the past two decades. The deterioration in health indicators in Romania during transition (increased incidence of mortality form cardiovascular diseases) is thought to be linked to a plethora of risk factors such as obesity [3]. Negative effects of fat consumption and positive effects of fruit/vegetables consumption on all causes mortality and premature death were reported [4]. Lifestyle factors (diet, cigarette smoking) compounded by economic hardship are thought to account for a large proportion of the disparity between life expectancy in the European Union (EU) and the Central and Eastern European countries (CEEC) [5-7]. Romania has received little attention as far as health and risk factors are concerned [8]. Policy makers need to understand how to motivate people to engage in preventive dietary behaviour. This paper draws upon a model of health preventive behaviour derived from social psychology literature and aims to identify the determinants of Dietary Health Preventive Behaviours (DHPB) in Romania and evaluate their impact in predicting DHPB.

Social cognition models have been used to understand health behaviour in developed economies [9]. The Health Belief Model (HBM) has received great research attention and numerous applications to health behaviours have been completed over the last three decades [10]. Nevertheless health motivation has been relatively overlooked in empirical studies [11].

**Figure 1:**
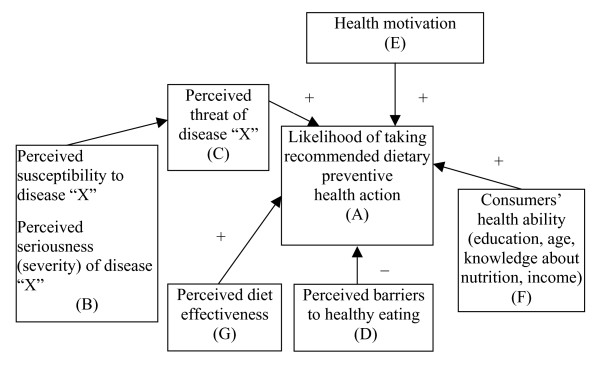
**The Romanian Dietary Health Preventive Behaviour Model**. Source: Adapted and extended from [11] and [12].

This study examines the applicability of the HBM (Boxes B and C in Figure 1) in the context of a transition economy by integrating it with key concepts from the theory of Health Preventive Behaviour [12] such as health motivation and health ability (Boxes E, F). Perceived diet effectiveness (Box G) was added to the model as an indicator of outcome expectancy.

Both the theory of Health Preventive Behaviour and HBM aim to explain the determinants of engaging in actions that can have health implications. There are two main assumptions underpinning the HBM: 1) the subjective valuation of a particular goal; and 2) the individual's estimate of the likelihood that a given action will achieve that goal [11]. The goals can be defined in terms of the prevention of disease or improvements to one's health status or wellbeing. According to the HBM (Figure 1), health behaviours (Box A) are dependent upon the perceived threat of disease (Box C). The latter is the outcome of perceived susceptibility to getting a disease and the severity of consequences of suffering the particular disease (Box B).

Given the importance of dietary factors in mortality in Romania, the focus of the study is the understanding of predictors of dietary preventive actions. The proposed Romanian DHPB model includes an adaptation of items to the Romanian context (Figure 1). The research design included the perceived threat of disease with high incidence that carry policy concerns in Romania [13]. The study incorporates perceived barriers to healthy eating (Box D Figure 1) identified in both western Europe [14], whilst retaining the weight of factors discussed in Romania [15].

## Methods

### Setting and subjects

The study was conducted in the capital of Romania in 2000 using random route sampling. Addresses were selected using the random route method following a stratification of the capital in 120 residential areas. A total of 485 face-to-face administrated questionnaires within the adult population took place at each respondent's residence. The sample was regarded as a minimum threshold to capture various segments of the Bucharest population and has been constrained by the financial resources available as part of the PHARE/ACE research project P97-9125-S. The project was approved by the Newcastle University Faculty of Agriculture and Biological Sciences Research Committee (ref CR/nf/9/03/2000).

Within each household the respondent was the individual responsible for household food shopping given that the extended questionnaire covered also this theme. The interviewers explained the purpose of the survey, ensured the anonymity and arrange a meeting convenient for the respondent. The interviews took place at respondent's residence following the verbal consent of participants. A small financial incentive was provided to participants. Finally, the authenticity of the questionnaires was tested by seeking confirmation from 10% of the respondents. The sample represented various segments of the population in the capital as follows: 31% were under 34 years old; 47% were aged between 35–54; 14.5% primary school leavers and 54.5% attended technical or high school. Women represented 77% of the sample.

### Measurements

Health behaviours have been defined as any action undertaken for the purpose of preventing disease or detecting it at an asymptomatic stage [16]. This study concentrates primarily on dietary health maintenance behaviours. Initially nine items related to DHPB (Box A, Figure 1) were included in the analysis guided by the theory [12] of health behaviour (items 1, 4, 6, 7, 8 and 9 in Table 1, appendix 1) and new items developed as a consequence of focus groups conducted in Romania (items 2, 3 and 5). All items were measured on a Likert five-point scale (1 = strongly disagree; 5 = strongly agree). Consistent with [12], the item related to alcohol moderation was treated as a single entity.

An Exploratory Factor Analysis (EFA) (principal component method) with varimax rotation led to the retention of seven items in highlighted italic font. Loadings above 0.40 were considered in the interpretation of factors, given the sample size [17].

Unidimensionality was assessed using Confirmatory Factor Analysis (CFA) in LISREL 8 [18]. For both dimensions items loaded positively as expected and with minimal cross-loading (Table 1), indicating unidimensionality [19]. The two-factor solution had a superior fit as indicated by the significant factor loadings (p < .05) and the goodness-of-fit indices (χ^2 ^= 8.43; df = 8; Root Mean Square Error of Approximation (RMSEA) = 0.011; Taka-Lewis Index (TLI) = 0.997; Adjusted Goodness-of-Fit Index (AGFI) = 0.984 relative to the null (one-factor solution) model (χ^2 ^= 39.83; df = 9). The first factor describes positive dietary actions, while the second describes negative dietary actions [12]. Each DHPB scale was derived as an arithmetic mean of the respondent's ratings corresponding to each of these two dimensions. The *information acquisition behaviour *was measured using a single item concerned with the frequency of using nutritional information from food labels: "How often do you read the information on food labels?" (1 = almost never; 5 = almost every time). *Health Motivation *(HM) is a central point in the theory of Health Preventive Behaviour [12]. HM was defined as the goal-directed arousal of consumers to engage in health preventive behaviour [20]. [21] linked the enduring motivation to the respondents' desire to process nutrition information in general and after exposure to a stimulus. Health motivation is regarded as consumer willingness to engage in preventive health behaviours (Box E, Figure 1). The original set of eight items [12,21] was reduced to six after piloting the questionnaire and discussions with health professionals (table 1, appendix 1). An EFA highlighted two dimensions of HM. The varimax rotation was selected to maximise the interpretability of factors (KMO = 0.66) which accounted for 62% of the variance. The communalities point out a significant proportion of variance (between 41% and 80%) of original variables explained by the complete set of derived factors. The first factor (items 10–13 in Table 1) describes a passive behaviour (Hmpassive) and a focus on short-term, hedonistic behaviour, which may be accounted by factors such as beliefs, personal values or a lack of awareness of health implications of dietary behaviour. The second factor (items 14–15 in Table 1) is concerned with actions undertaken to prevent the onset of health problems, describing a proactive behaviour (Hmactive).

The two-factor solution generated by the EFA has been validated by the CFA. The predicted two-factor solution had a superior fit as indicated by the significant factor loadings (p < .05) and the goodness-of-fit indices (χ^2 ^= 17.82; df = 8; RMSEA = 0.05; Taka-Lewis Index 0.971; AGFI = 0.968) relative to the null model (χ^2 ^= 179.55; df = 9), which specified a single factor solution. Hence, unlike [12], the items were not retained as a single construct, but regarded as a two-factor solution.

Severity of disease (Box B, Figure 1) was measured by asking respondents to evaluate how disturbing they found specific prompted diseases (1 = not disturbing at all; 5 = very disturbing). Susceptibility to disease was measured with a perceived likelihood to suffer from prompted diseases during the following five years (1 = very unlikely to 5 = very likely). The multiplicative combination of these two scales (severity and susceptibility) yields the "*Perceived Threat*" score (Box C, Figure 1) corresponding to each disease. Five diseases were prompted as follows: high blood pressure, ulcer, liver disease, diabetes and ischaemic heart disease (IHD). The overall threat score represents an arithmetic mean of the five scores corresponding to each disease.

*Perceived Barriers to Healthy Eating *(Box D, Figure 1). Respondents were asked whether barriers on a list impinge on their attempts to pursue healthy diets (yes/no questions). The items used for eliciting answers related to the perception of barriers to a healthier diet were derived from the consumer behaviour literature and studies concerned with food choice [14,22]. One specific item was added, namely "the pressure on my diet", as it was felt that consumers in Romania face significant budgetary constraints that impinge upon their food choices. It referred to financial constraints on dietary choices. The barriers are reported in table 2.

Regarded a*s *"consumers resources, skills, or proficiencies for performing preventive health behaviours" [[12], p.210] *health ability *(Box F, Figure 1) is measured with four dimensions: education, age, income and knowledge about nutrition.

Education, measured as an average number of years of schooling was calculated (8 = primary school; 10 = technical school; 12 = high school; 16 = university graduates).

The Observed Knowledge about Nutrition (KNO) was measured on 11 dichotomy true/false (T/F) items. Items were carefully mixed so that the likelihood of guessing the right answer was substantially reduced. Items from [23] were combined with new items (e.g. item 7) generated from the focus groups conducted in Romania (Table 3).

An important assumption of the nutrition knowledge scale is that the items used in the scale development reflect the information required by the individuals to make dietary choices [24]. The nutrition knowledge scale encapsulated all three types of knowledge set out by [25]: awareness of diet and disease relationships (items 4–5), knowledge of principles of nutrition (items 7–8), knowledge of food nutrient density (items 1, 3, 6 and 9).

The difficulty factor was given by the percentage of correct responses in the sample. Only the items with a difficulty factor between 25% and 75% were maintained. This ensured that the items generated an acceptable discriminant capacity. The six items retained in the analysis (outlined in italic font) provided a satisfactory coefficient of reliability: Kuder Richardson (KR20) = 0.63 [26]. An additive measure of knowledge about nutrition for each respondent is included in the model.

A Self-reported measure of consumer Nutritional Knowledge (KNS) was also included (1 = not very knowledgeable at all; 5 = very knowledgeable). As a subjective measure of knowledge may contain a social desirability bias [27] it was useful to measure both a self-reported and objective measure of the same latent construct.

Income is approximated by the self-reported financial status of the respondents household was measured on an ordinal scale (1 = very difficult; 6 = very good).

*Perceived diet effectiveness *(Box G) is measured as the strength of the respondent's beliefs that disease can be prevented through an adequate diet (1 = strongly disagree; 5 = strongly agree). Three groups of diseases (cancer, heart-related, liver-related) were prompted.

### Psychometric properties of constructs

A summary of the internal reliability (inter-item consistency) and convergent validity derived from the CFA of the constructs can be found in Table 4. Values of composite reliability greater than 0.7 for established scales and 0.6 for new scales are indicative of internal consistency. Values of Average Explained Variance (AVE) greater than 0.5 indicate convergent validity [28].

Only one variable (Negative actions) is well below the threshold. The squared root of AVE for each construct tended to exceed the correlation of the specific construct and the other constructs indicating discriminant validity [29]. [30] points out that one-third to two-thirds of the variance in a typical consumer research measure can be due to measurement errors. An internal consistency reliability coefficient of 0.27 to 0.87 was reported in previous studies related to health practices [31]. Most scales with the exception of negative diet actions satisfy the minimum recommended standards.

All loadings were statistically significant and LISREL indices provide evidence of adequate fit and showed evidence of discriminant validity. The factor solutions validated by the CFA provide supportive evidence of construct validity. Given the marketing and health policy challenges of health behaviour, both dimensions of DHPB will be analysed.

## Hypotheses

In line with the HBM predictions, the anticipated severity of disease and perceive susceptibility to disease have been reported to have positive effects on precaution measures [11] whereas perceived barriers to preventive action has a negative effect. Mixed support for the role of health motivation on health preventive behaviour has been found [12]. It has been pointed out that nutritional knowledge and the level of education will reflect the ability of individuals to process health and diet-related information. Respondents with higher education levels are more likely to use nutrition information from food labels [32,33].

Consumer age influences consumer "mental and physical ability to select and implement health behaviours" [[12], p. 210]. Consumer income reflects the financial ability to implement health concerns in dietary choices. Perceived diet effectiveness, as a measure of action-outcome expectation [34], is expected to have a positive effect on DHPB. Hence it is proposed that:

H1: Consumers with higher perceived threat of disease (Box C) will be more likely to engage in DHPB (Box A).

H2: Consumers with higher levels of perceived barriers to healthy eating (Box D) will be more likely engage in DHPB (Box A).

H3: Consumers with higher health motivation (Box E) will be more likely engage in DHPB (Box A).

H4: Consumers with higher health ability (namely knowledge about nutrition, education level, age and income) (Box F) will be more likely to engage in DHPB (Box A).

H5: Consumers with stronger beliefs that diet can prevent disease (diet effectiveness) (Box G) will be more likely to engage in DHPB (Box A).

Modifying factors such as age, sex, personality can impact on the perceived susceptibility/seriousness of disease [35]. There is an expectation that women are more health-conscious and interested in health issues and reported more frequently in information seeking behaviour [31], hence more likely to be aware of health hazards. With regards to age, [36] pointed out that younger subjects may be less realistic about future health problems.

H6: Women will be more likely than men to perceive a high susceptibility to disease.

H7: Younger respondents will be less likely than older respondents to perceive a high susceptibility to disease.

The following section reports on the testing of the hypotheses using the survey data collected in Romania.

## Results

### Perceived health hazards

Tables 5 and 6 report on the significance of characteristics of respondents on their perceived susceptibility to disease.

Overall, all demographic variables are significantly associated with the perceived susceptibility to disease. However, their significance varies according to each specific disease. Namely, women tend to be more aware of the risk of liver disease. This is consistent with the higher optimism regarding the own health status reported by men in Romania [3].

A higher proportion of young people believe they are unlikely to experience health hazards such as blood pressure or heart disease. The relationship between susceptibility to disease and education is equivocal. [37] reported that optimistic bias is unrelated to age, sex, education or occupational prestige.

### Antecedents of DHPB

Table 7 reports the multivariate results of the theory test. Monetary income has been eliminated from the model given the large proportion of non-responses (45.2%). Against a background of a significant shadow economy [38], people are unwilling to reveal their actual income. The model includes an ordinal scale of self-reported financial status, as it was perceived less intrusive and generated a higher response rate.

The general to particular approach in model selection [39] was followed. All the variables specified in the conceptual model were included in a first stage. In subsequent stages variables with non-significant betas were removed one at a time.

Independent variables were mean centered to reduce collinearity. The latter was unproblematic, given the Variance Inflation Factors (VIF) [40] and reasonable values of correlations among independent variables (.01–.45 with most variables correlated at ρ<.15).

The explained variation in some models is modest, which is unsurprising as the joint effects of independent variables are typically low in HBM applications [10]. Nevertheless R^2 ^values are comparable to the literature [12].

Respondents with higher levels of health motivation, stronger beliefs that diet can prevent disease and higher levels of knowledge about nutrition, are more likely to perform DHPB. Respondents age and education play also a positive role. Contrary to theoretical expectations, perceived threat of disease has a negative effect on the decision to engage in DHPB.

The frequency of engaging in health information-acquisition behaviours such as reading food labels is positively predicted by informants' health motivation (hmactive), income, the level of formal education and particularly by the KNS. Age is also a predictor but its effect is negative.

## Discussion

A significant segment of respondents believed they are not susceptible to the elicited diseases. Health promotion strategies should aim to change the judgments of health risk. The structure of message cues can impact upon perception of risk. [41] argued that using a list of a greater number of frequent behaviours preceding a lesser number of infrequent behaviours can increase involvement and perceived vulnerability.

Some evidence against H1 was found. Perceived threat of disease emerges with a negative sign contradicting theoretical expectations. It is possible that perceived locus of control may contribute to this pattern by acting as a moderating variable. Of the prompted diseases perceived threat diabetes has a stronger negative effect on DHPB. Many respondents who attribute this condition to genetic predispositions may initiate little preventive action if they hold strong believes that they are either immune to or prone to such illness, this kind of behaviour being reinforced by a fatalistic feature of Romanian culture [42].

Perceived threat has generally been found to be less significant in HBM applications [43]. Even in developed countries perceived threat has been regarded as a secondary predictor of behaviour [34]. The susceptibility to get a disease is not a significant driver of consumer behaviour, probably because most Romanian consumers under the pressure of low-incomes tend to trade off long-term health benefits for short-term benefits (lower prices). Economic factors have been reported as a significant barrier to food consumption [15]. Table 2 shows that economic and psychological barriers (consumer preferences) are the most frequent in Bucharest. Some of the barriers are not easy to be addressed (low income). However, other barriers are more controllable by individuals or can be influenced by marketers (changes in consumer preferences).

Almost a quarter of respondents suffering from disease are unable to state in the survey whether it is linked to diet. There is therefore scope to increase the awareness of diet-disease relationships with potential gains in terms of dietary behavioural change, as highlighted by the estimate of perceived diet effectiveness.

Partial evidence in support of H2 was found. Perceived barriers to healthier eating emerges as a significant predictor only in the case of alcohol moderation. The more barriers that are perceived the less likely someone will moderate the alcohol intake. This results raises policy concerns as alcohol is sometimes used as a stress coping mechanism. A high incidence of alcoholism (linked to spirits consumption) in transitional economies has been linked to diseases such as cirrhosis [44].

The likelihood of engaging in DHPB is greater when people are highly motivated with respect to health (H3) and there is a stronger belief that diet can prevent disease (H5).

Evidence of a positive influence of age and knowledge about nutrition on DHPB was found. The respondent's level of education positively influenced positive dietary actions and information acquisition behaviours.

There is mixed evidence of the role of heath ability (H4) on performing DHPB. Similarly to [9] KNO is a positive predictor of positive diet, while age has a positive effect on negative diet and negative effect on information acquisition behaviour.

Higher levels of education are associated with higher levels of information search [45] and acquisition of nutrition information [32,46,47].

The impact of nutrition knowledge on food label use has generated little agreement in the literature. While [48] argued that nutrition knowledge influences consumer ability to perform tasks related to nutrition labels, [49] found no relationship between knowledge and labels use. Our study is in line with the latter study.

In Romania food labels have not been well promoted as a vehicle of nutrition and health messages. Under a legacy of food shortage, consumers had scarce opportunities to develop habits of using labels. It is the self-reported measure of nutrition knowledge that influences label use. This measure may simply reflect confidence in own knowledge rather than actual knowledge.

It is not excluded that the confidence related to nutrition knowledge may disguise a spurious relation with label use. [21] highlighted that although age is associated with self-reported ability to process nutrition information, it is negatively linked to nutrition label comprehension.

The role of age is equivocal. While age is positively associated with DHPB, there is a negative association with information acquisition which corroborates this [12]. The latter finding may be linked to the small font size of nutritional facts information on many food labels in Romania. [32] found that the probability of using nutrition information from labels decreases with age.

However explanations of less information processing among the elderly [50] attributable to greater market experience have limited validity in the Romanian post-communist context. During the market liberalisation many new products and brands were introduced and consumers are in a learning curve. Factors such as the limited ability to read and comprehend labels are not excluded particularly in this segment. Food labels in Romania are not well regulated relative to Western Europe creating frequent problems in the comprehension of nutritional information. Thus elderly, despite the arguably higher need for special diets typically associated with increased likelihood of using the nutritional information [51,33] rely actually less on food labels in Romania.

There is some debate over the role of income in influencing nutrition information search. Income tends to be associated with high levels of education, health consciousness and nutrition knowledge [33] conducive to high levels of search behaviour. Yet income in the developed world is also associated with time pressure constraining the amount available for in-store decisions and hence limiting the information search initiatives [49].

The Bucharest study points to a positive impact of self-reported financial status on information search. A higher demand for information is apparent among the better-off. Given the average low levels of real incomes in Romania it is unsurprising that lower income respondents can hardly afford to incorporate health and nutrition information in their dietary choices.

Although it is found that age influences the likelihood to engage in DHPB, food label use represents a contrasting case. The ageing process is arguably associated with higher awareness of health and diet-disease relationships [51], stronger perceived diet effectiveness [52]. Yet, the ability to read nutrition information from labels can be reduced by factors such as visual impairment.

Some evidence if favour of H5 was found. Respondents who believe in diet effectiveness are more likely to be engaged in DHPB, but the effects on alcohol moderation and information search were insignificant.

Health motivation emerges as the most significant predictor of health information acquisition and health preventive behaviour. In contrast to [12] it is Hmactive that emerged as a significant factor across all equations. Moreover, this variable predicted also alcohol moderation. The motivation to engage in healthful behaviour was found a significant determinant of the likelihood to make lifestyle changes [53].

Surprisingly no significant gender-related differences in terms of the likelihood of engaging in DHPB were found. There is an expectation that women are more health-conscious and interested in health issues [31]. The higher awareness of health hazards among women in Bucharest is not necessarily translated into a more active engagement in DHPB. The barriers that do not allow women to engage more in DHPB need to be further explored.

Although all demographic variables are significantly associated with perceived susceptibility to illness, the significance varied according to disease. Only susceptibility to liver disease is significantly associated with gender. Therefore the evidence behind H6 is weak. Younger respondents believe they are less likely to suffer liver disease, heart disease and high blood pressure. There is therefore partial evidence in support of H7. This study partly corroborates previous research [36].

## Conclusion

Drawing from the health behaviour literature, this article sought to identify predictors of consumer dietary health preventive behaviour. Respondents' health motivation, perceived diet effectiveness, knowledge about nutrition, education and age were significant positive predictors of DHPB as expected. The information acquisition behaviour is positively predicted by health motivation, education, self-reported financial status and knowledge about nutrition and negatively by age.

In contrast to theoretical expectations, perceived threat of disease exerts a negative influence on DHPB. Health campaigns therefore need to increase the perceived link between the behavioural plan and future health outcomes.

The role of income in influencing DHPB remains ambiguous. Although economic factors appear on the top of perceived barriers to healthier eating, income appeared statistically insignificant in three regression models. It is not excluded that many high-income earners are not engaged in DHPB to the extent noticeable in developed countries, given that the wealth was acquired in a relatively short period of time. However, there is evidence that low-income earners perceive economic barriers as impediments to their ability to eat healthier. The role of monetary income in influencing DHPB deserves further research given the significant non-responses in this study and the inconclusive role in other studies [12].

Surprisingly, it was the self-reported rather than actual knowledge about nutrition that predicted the alcohol moderation and the information acquisition behaviour from food labels. This pattern suggests that self-confidence in nutrition knowledge may not always mirror the actual knowledge about diet. It is not excluded that some respondents may have overstated reading information from food labels (social desirability effect).

The literature records little agreement over the role of nutrition knowledge in health prevention. Knowledge about diet is not necessarily translated into healthier choices, as factors such as strong preferences for unhealthy foods and entrenched eating habits, family preferences [14] may inhibit such choices. If the knowledge about nutrition of the head of the household (responsible for the food purchases) is poor, then the adverse effects on diet will be experienced by almost all family members. Family preferences were reported as barriers to healthy eating. Media campaigns concerned with healthy eating may be oriented towards the family rather than individuals, given their social and economic vulnerability [54]. Tailored nutrition messages may be effective in changing food practices [55].

Nutrition education campaigns have had mixed success [41] in shaping consumer dietary choice. Notwithstanding the difficulty of changing eating habits, this study adds further support to the need for targeting in elaborating health and nutrition campaigns. There is a significant proportion of informants who believe they are not at risk of disease. As self-positive bias hinders message processing [37,41], greater effort should be placed in media strategies aimed at changing people's perceptions about hazards targeted at this group (predominantly young subjects and people with lower education levels).

The study provides insights for future campaigns concerned with promoting healthy eating. Educational campaigns may therefore consider youth and subjects with low levels of education and knowledge about nutrition as a primary targets. Marketing campaigns promoting healthier dietary choices (e.g. low-fat foods) in this region can target consumers with high levels of health motivation and education. There is scope for changing social perceptions of healthy eating by promoting meal solutions that combine healthiness with palatability.

Drawing from the UK experience of healthy eating campaigns which are part of the Health of the Nation strategy, there is scope for both increasing the awareness of links between health, wellbeing and specific foods. [56] reported a lack of information about health behaviours and a low awareness of the relationships between lifestyle factors and risk of CVD in Eastern Europe compared to Western Europe. Our study emphasises the need to consolidate this awareness, narrowing the gap between Eastern and Western Europe.

Of particular policy concern is that elderly, who are more likely to have special dietary requirements, are not using information from food labels. This can be linked to the legacy of little exposure to and experimentation of well-elaborated labels during the communist era compounded by the proliferation of many hardly comprehensible food labels during the market liberalisation. Moreover packaged food tends to be available at a premium and is perhaps beyond the reach of consumers with modest incomes, particularly the retired where the incidence of poverty is high [57].

In the light of survey results, health-related messages stressing perceived vulnerability may not be effective. It has been reported that risk acceptability may play a moderating role [58] in risk perceptions. Positive campaigns focused on benefits of dietary change (what should be eaten) may be more suitable, particularly in the early stages of behavioural change [59]. There is scope for improving consumer knowledge about dietary fat and cholesterol particularly in the context of high incidence of CVD.

Several limitations of the study should be considered when interpreting the results. First, the conclusions are valid only for the capital Bucharest. Significant differences in consumer behaviour between urban and rural areas in Romania [60] may impact on the importance of predictors of DHPB. Second, a more detailed set of variables (e.g. perceived behavioural or health locus of control) would provide a more extensive picture of antecedents of DHPB.

## Competing interests

The author(s) declare that they have no competing interests.

## Authors' contributions

DAP designed the survey, analysed the data and drafted the paper. CR advised on the structure and content of the questionnaire and commented on the drafts of the paper.

Both authors read and approved the final manuscript.

## Pre-publication history

The pre-publication history for this paper can be accessed here:



## Figures and Tables

**Table 1:** A summary of selective study construct measures.

**Construct**	**Items used**	**Factor loadings**
Dietary Health Preventive Behaviour	Positive actions	(.746)
	1. *I try to consume a lot of fruit and vegetables*	.84
	2. *I choose products rich in fiber*	.54
	3. *I often choose products with low animal fat content*	.59
	Negative actions	(.716)
	4. *I try to eat as little salt as possible*	.59
	5. *I try to avoid stimulants (coffee, cola)*	.74
	6. *I try to have a balanced diet*	.54
	7. I try to avoid confectionery products.	
	8. I do not eat some foods because they make me fat.	
	Alcohol moderation	
	9. *I try to consume alcohol with moderation*	

Health motivation	Passive behaviour (Hmpassive)	(.712)
	10. I do not change my behaviour until I do have a health problem.	.46
	11. I would rather enjoy life than avoiding exposing myself to all health hazards.	.96
	12. I don't worry about health hazards until there appear diseases that become a problem.	.59
	13. I am not going to worry about all the problems as there are so many problems that can hurt you these days.	.62
	Proactive behaviour (Hmactive)	(.783)
	14. I am concerned about health hazards	.67
	15. I try to prevent health problems	.74

Note: Construct/composite reliabilities are reported in parentheses

Source: derived from authors survey data

**Table 2:** Percentage of respondents who reported barriers to healthier eating practices.

	% of respondents
It is hard to give up to the food you like	78
High price of healthy food	78
Pressure on my budget	70
Healthy food not available in stores	40
Lack of time	34
Nutritionists change their opinions	30
Lack of knowledge about healthy eating	30
Weak endowment with kitchen equipment	29
Lack of understanding of family members	22

Source: authors survey data

**Table 3:** The nutrition knowledge scale.

Knowledge about health and nutrition	Difficulty factor (% of respondents answering correctly)
1. *Whole milk is a better source of calcium than skimmed milk. F*	39.4
2. *Removing the skin from chicken reduces the fat content. T*	68.8
3. *Eating more bread helps to increase protein in the diet. F*	49.2
4. *Any type of fat may damage the health. F*	39.6
5. A high intake of salt may increase blood pressure. T	87.7
6. Butter contains more cholesterol than margarine. T	87.9
7. *The daily calorie intake should not exceed in general 3200. T*	70.0
8. *No more than a third of calories should come from fat. T*	58.6
9. White bread is more nutritious than wholemeal bread. F	87.8
10. Soya beans are a good source of proteins. T	80.1
11. Cholesterol is found only in foods containing fat or oil. F	22.1

Source: authors survey data

**Table 4:** Psychometric Properties of Constructs.

Construct	Items	Mean	SD	Reliability diagnosis	AVE
A. Health behaviours					
Positive diet action	3	3.6	0.7	0.6	0.34
Negative diet action	3	3.4	0.7	0.5	0.25
Alcohol moderation	1	3.6	1.2	...	...
Label information acquisition	1	3.9	1.1	...	...

B. Health motivation					
Proactive action	2	3.7	0.7	0.78	0.65
Passive behaviour *	4	2.5	0.7	0.71	0.39

C. Health ability characteristics					
Nutrition knowledge scale	6	...	...	...	...
Self-reported nutrition knowledge	1	3.4	0.9		
Education	1	12.5	2.7	...	...
Age	1	43.4	15.1	...	...
Income (sterling pounds)	1	77.6	68.3	...	...

D. Perceived threat of disease	5	7.6	5.2	0.82	0.49
E. Efficacy	3	4.1	0.6	0.86	0.68

NOTE: The composite or construct reliability is evaluated based on Cronbach's alpha for continuous variables: (∑λ_*i*_)^2^/(∑λ_*i*_)^2 ^+ (∑v [δ_*i*_]). The convergent validity is evaluated using the average variance extracted: AVE = (∑λ_*i*_^2^)/(∑λ_*i*_^2^) + (∑v [δ_*i*_]); where λ_*i *_= completely standardised factor loading for item i; ∑v [δ_*i*_] = completely standardised error variance for item i.

*) Passive behaviour: the scale values were reversed so that high scores reflect high levels of health motivation.

Source: own calculations

**Table 5:** Demographic predictors of susceptibility to disease.

	Incidence % of sample^1^	Chi-square statistics^2^		
		**Gender**	**Education**	**Age**
Blood pressure	23	0.17	1.48	5.90*
Liver diseases	22	2.70++	5.80*	8.7*
Diabetes	11	0.53	6.55++	0.67
Ulcer	18	3.24	2.10	4.20
Ischaemic heart disease	21	0.92	2.97	5.27++

Note: ^1 ^Proportion of respondents who answered 4 and 5 on a five-point scale regarding susceptibility to disease. ^2 ^Observed values of crosstabs of respondents grouped by susceptibility to disease (below/above median score) and demographics.

Source: derived from authors survey data

**Table 6:** Proportion of respondents with above median scores on susceptibility to disease among total demographic subgroup.

	**Gender**		**Education**				**Age**		
	M	F	Primary school	Technical	High School	Univ.	<34	35–54	>55
Blood pressure	56.5	53.4	61	45.5	55.9	53.8	46.1	60.3	62.5
Liver diseases	43.1	55.2	63.6	60.7	44.2	61	45.8	61.4	50
Diabetes	22.1	26.5	36.2	20	22.1	33.7	30.3	27.4	24.6
Ulcer	34.4	47.4	51.2	40.7	43.3	51.1	47.6	51.1	35.5
IHD	37.3	43.9	56	40.7	42.8	44	36.4	47.6	53.1

Note: ** p < .01; * p < .05; ++ p < .10

Source: derived from authors survey data

**Table 7:** Regression analysis of antecedents of DHPB.

	Positive diet action	Negative diet action	Alcohol moderation	Food label use
Health Motivation active	0.11*	0.22**	0.11**	0.16**
Health Motivation passive	0.09*		0.07+	
Perceived Diet Effectiveness	0.16**	0.24**		
Nutrition Knowledge (KNS)	0.11*		0.08++	0.31**
Nutrition Knowledge (KNO)	0.09*			
Age		0.14**		-0.08+
Education	0.07+			0.11**
Perceived Threat of disease	-0.14**	-0.10*		
Perceived Barriers			-0.11++	
Self-reported financial status				0.13**

N	435	457	468	430
R^2^	0.12	0.18	0.05	0.20
Mean VIF	1.05	1.04	1.02	1.11

Notes: Only significant standardised predictors are reported. All univariate tests are significant. ** p < .01; * p < .05; ++ p < .10; +p < .15

Source: derived from authors survey data

## References

[B1] NIS (National Institute of Statistics) (2002). Anuarul Statistic al Romaniei.

[B2] Cockerham WC (1999). Health and social change in Russia and Eastern Europe.

[B3] NIS (2000). Health status of the population in Romania.

[B4] Arah OA, Westert GP, Delmoij DM, Klazinga NS (2005). Health system outcomes and determinants amenable to public health in industrialized countries: a pooled, cross-sectional time series analysis. BMC Public Health.

[B5] Bobak M, Marmot M, Hertzman C, Kelly S, Bobak M (1996). East-West health divide and potential explanations. East-West Life Expectancy Gap in Europe: Environmental and Non-environmental Determinants.

[B6] Hertzman C, Kelly S, Bobák M, (Eds.) (1996). East-West life expectancy gap in Europe: Environmental and non-environmental determinants.

[B7] Petrovici DA, Ritson C Population, health and risk factors in a transitional economy. J Consumer Policy.

[B8] Dolea C, Nolte E, McKee M (2002). Changing life expectancy in Romania after the transition. J Epidemiology Community Health.

[B9] Rutter D, Quine L, Rutter D, Quine L (2002). Social cognition models and changing health behaviours. Changing Health Behaviour: Intervention and Research with Social Cognition Model.

[B10] Sheeran P, Abraham C, Conner M, Norman P (1996). The health belief model. Predicting Health Behaviour.

[B11] Janz NK, Becker MH (1984). The health belief model: a decade later. Health Education Quarterly.

[B12] Moorman C, Matulich E (1993). A model of consumers' preventive health behaviour: the role of health motivation and health ability. J Consumer Research.

[B13] Palmer S, Poledne R, Carroll KK (1998). Perspectives from Eastern Europe. Current Perspectives on Nutrition and Health.

[B14] Lapallainen R, Saba A, Holm L, Mykkanen H, Gibney MJ (1997). Difficulties in trying to eat healthier: descriptive analysis of perceived barriers for healthy eating. European Journal of Clinical Nutrition.

[B15] Stanculescu M (1999). Saracia in Romania: 1990–1998.

[B16] Kasl SV, Cobb S (1966). Health Behaviour, Illness Behaviour and Sick Role Behaviour. Archives of Environmental Health.

[B17] Hair JF, Anderson RE, Tatham RL, Black WC (1998). Multivariate Analysis with Readings.

[B18] Jöreskog KG, Sörbom D, Du Toit S, Du Toit M (2001). LISREL 8: New Statistical Features, SSI.

[B19] Anderson J, Gerbing D (1988). Structural equation modelling in practice: A review and recommended two-step approach. Psychological Bulletin.

[B20] MacInnis DJ, Moorman C, Jaworski BJ (1991). Enhancing and measuring consumers' motivation, opportunity, and ability to process brand information from ads. J Marketing.

[B21] Moorman C (1990). The effects of stimulus and consumer characteristics on the utilisation of nutrition information. J Consumer Research.

[B22] Asp E (1999). Factors affecting food decisions made by individual consumers. Food Policy.

[B23] Alexander JM, Tepper BJ (1995). Use of reduced-calorie/reduced-fat foods in young adults: influence of gender and restraint. Appetite.

[B24] Axelson ML, Brinberg D (1992). The measurement and conceptualization of nutrition knowledge. J Nutrition Education.

[B25] Blaylock JD, Smallwood K, Kassel J, Variyam AL (1999). Economics, food choice, and nutrition. Food Policy.

[B26] Lewis-Beck MS (1994). Basic measurement International handbooks of quantitative applications in the social sciences.

[B27] Palmer RF, Graham JW, Taylor B, Tatterson J (2002). Construct validity in health behaviour research: interpreting latent variable models involving self-report and objective measures. J Behavioural Medicine.

[B28] Fornell C, Larcker DF (1981). Evaluating structural equation models with unobservable variables and measurement errors. J Marketing Research.

[B29] Gefen D, Straub D (2005). A practical guide to factorial validity using PLS-graph: tutorial and annotated example. Communications of the Association for Information Systems.

[B30] MacKenzie S (2001). Opportunities for improving consumer research through latent variable structural equation modelling. J Consumer Research.

[B31] Rakowski W, Assaf AR, Lefebvre RC, Lasater TM, Niknian M, Carleton RA (1990). Information-seeking about health in a community sample of adults: correlates and associations with other health-related practices. Health Education Quarterly.

[B32] Kim S, Nayga RM, Capps O (2001). Food label use, self-selectivity, and diet quality. J Consumer Affairs.

[B33] Drichoutis CA, Lazaridis P, Nayga MR (2005). Nutrition knowledge and consumer use of nutritional food labels. European Review of Agricultural Economics.

[B34] Conner M, Sparks P, Conner M, Sparks P (2002). The theory of planned behaviour and health behaviours. Predicting Health Behavior Search and Practice with Social Cognition Models.

[B35] Glanz K, Rimer BK, Lewis FM (2002). Health Behaviour and Health Education Theory, Research and Practice.

[B36] Johnson RJ, McCaul KD, Klein WMP (2002). Risk involvement and risk perception among adolescents and young adults. J Behavioural Medicine.

[B37] Weinstein ND (1987). Unrealistic optimism about susceptibility to health problems: conclusions from a community-wide sample. J Behavioural Medicine.

[B38] EIU (The Economist Intelligence Unit) (2003). Romania: Country report.

[B39] Hendry DF, Mizon GE (1978). Serial correlation as a convenient simplification, not a nuisance: a comment on a study of the demand for money by the Bank of England. The Economic Journal.

[B40] Mason CH, Perraut WD (1991). Collinearity, power and interpretation of multiple regression analysis. J Marketing Research.

[B41] Menon G, Block LG, Ramanathan S (2002). We're at as much as we are led to believe: effects of message cues on judgments of health risks. J Consumer Research.

[B42] Vulcanescu M (1991). Dimensiunea româneasca a existentei.

[B43] Yates JF (1992). Risk-taking behaviour.

[B44] McKee M, Shkolnikov V (2001). Understanding the toll of premature death among men in eastern Europe. British Medical Journal.

[B45] Schultz WT (1975). The value of the ability to deal with disequilibria. J Economic Literature.

[B46] McLean-Meyinsse PE (2001). An analysis of nutritional label use in the Southern United. States. J Food Distribution Research.

[B47] Nayga MR (1996). Determinants of consumers' use of nutritional information on food packages. J Agricultural and Applied Economics.

[B48] Levy SA, Fein BS (1998). Consumers' ability to perform tasks using nutrition labels. J Nutrition Education.

[B49] Nayga MR (2000). Nutrition knowledge, gender and food label use. J Consumer Affairs.

[B50] Phillips L, Sternthal B (1977). Age differences in information processing: a perspective on the aged consumer. J Marketing Research.

[B51] Senauer B, Asp E, Kivey J (1991). Food trends and the changing consumer.

[B52] Senesi SI, Nayga RM, Gómez G, Palau H, Ordoñez HA (2006). Nutrition knowledge and consumer use of nutritional labels. International Food & Agribusiness Management Association, World Food & Agribusiness Symposium, Argentina.

[B53] Ferrini R, Edelstein S, Barrett-Connor E (1994). The association between health beliefs and health behavior change in older adults. Prev Med.

[B54] MacLean WP, Gittinger JP, Leslie J, Hoisington C (1987). Nutritional risk: concepts and implications. Food policy: integrating supply, distribution, and consumption.

[B55] Brinberg D, Axelson ML (2002). Improving the dietary status of low-Income pregnant women at nutritional risk. J Public Policy & Marketing.

[B56] Steptoe A, Wardle J (2001). Health behaviour, risk awareness and emotional well-being in students from Eastern and Western Europe. Social Science and Medicine.

[B57] World Bank (1996). Romania: poverty and social policy.

[B58] Rindfleisch A, Crocket DX (1999). Cigarette smoking and perceived risk: a multidimensional investigation. J Public Policy & Marketing.

[B59] Ling AMC, Horwath C (2001). Perceived benefits and barriers of increased fruit and vegetable consumption: validation of decisional balance scale. J Nutrition Education.

[B60] Manrai L, Lascu DN, Manrai A (2000). How the fall of the Iron Curtain has affected consumers' perceptions of urban and rural quality of life in Romania. J East-West Business.

